# Editorial Comment: Flexible Ureterorenoscopy Versus Shockwave Lithotripsy for Kidney Stones ≤ 2 cm: A Randomized Controlled Trial

**DOI:** 10.1590/S1677-5538.IBJU.2022.06.04

**Published:** 2022-11-01

**Authors:** Alexandre Danilovic

**Affiliations:** 1 Universidade de São Paulo Hospital das Clinicas Faculdade de Medicina Departamento de Urologia São Paulo SP Brasil Departamento de Urologia, Hospital das Clinicas Faculdade de Medicina da Universidade de São Paulo – FMUSP, São Paulo, SP, Brasil; 2 Hospital Alemão Oswaldo Cruz São Paulo SP Brasil Serviço de Urologia, Hospital Alemão Oswaldo Cruz, São Paulo, SP, Brasil

Andrea Bosio ^1^, Eugenio Alessandria ^2^, Ettore Dalmasso ^2^, Simone Agosti ^2^, Federico Vitiello ^2^, Eugenia Vercelli ^2^, Alessandro Bisconti ^2^, Paolo Gontero ^2^

**Eur Urol Focus. 2022:21;S2405-4569(22)00081-5**

**DOI: 10.1016/j.euf.2022.04.004 | ACCESS: 35466071**

## COMMENT

Bosio et al. conducted a randomized controlled trial to compare the outcomes of flexible ureterorenoscopy (RIRS) versus shockwave lithotripsy (SWL) for kidney stones up to 2 cm. The primary endpoint was stone-free rate (SFR) at 1 month (1). Secondary endpoints were SFR at 6 months and 1 year, complications and secondary treatments. True SFR (zero residual fragments) at 1 month favors RIRS (50.0% vs. 26.5%; p=0.004). In the long run, the authors state that this difference tends to reduce at 6 months 59.4% vs. 40.9%; p=0.032 and no longer be significant at 1 year 55.9% vs. 48.4%; p=0.392 (1). Although it is very difficult to control variables for 1 year, authors suggests that clearance of residual fragments after SWL persists longer than after RIRS. Clearance rate of residual stone fragments ≤ 2 mm after RIRS is about 80% during the first 3 months whereas less than 12% of the residual stone fragments > 2mm (2). Another important point is that patients in the SWL arm were submitted to further treatments during one-year follow-up more than patients in the RIRS arm, including flexible ureterorenoscopy, which could had artificially increased the success of shockwave lithotripsy.

This study also compared the outcomes of each treatment from the patient point of view. 87.1% of the patients of the RIRS arm vs. 88.2% of the SWL arm; p=0.845 were satisfied with their own treatment. Despite these good satisfaction rates, there is room for improvement. Patients would not choose the same treatment again in 22.9% of RIRS arm vs. in 13.2% of SWL arm; p=0.142. Patients of the RIRS arm that would choose another treatment reported stent-related symptoms as the reason in 56.3% and patients of SWL arm reported negative outcome in 77.7% (1). Improvements in ureteral stents and better patient selection seem to be the keys to increase patient's satisfaction with the treatment.

One limitation of this study is the image evaluation. Only stones 16-20 mm on ultrasound (US) and kidney-ureter-bladder X-ray (KUB) preoperative evaluation were submitted to computed tomography (CT) and none postoperative image evaluation used CT. Although CT is the gold standard image modality to determinate true SFR and US + KUB are not a reliable tool to identify residual stone fragments 0-2 mm, authors used only US + KUB for both groups (2).

To sum up, SWL remains a viable alternative for the treatment of kidneys stones, particularly for selected patients. Also, every effort should be made at the time of flexible ureterorenoscopy to leave no stone fragment behind and to maintain high SFR. Mitigation of stent-related symptoms is crucial to increase patient's satisfaction with RIRS.
